# Choline Supplementation Prevents a Hallmark Disturbance of Kwashiorkor in Weanling Mice Fed a Maize Vegetable Diet: Hepatic Steatosis of Undernutrition

**DOI:** 10.3390/nu10050653

**Published:** 2018-05-22

**Authors:** Thaddaeus May, Kevin C. Klatt, Jacob Smith, Eumenia Castro, Mark Manary, Marie A. Caudill, Farook Jahoor, Marta L. Fiorotto

**Affiliations:** 1 Childrens’ Nutrition Research Center, Department of Pediatrics, Baylor College of Medicine, 1100 Bates Street, Houston, TX 77030, USA; ecastro@bcm.edu (E.C.); fjahoor@bcm.edu (F.J.); martaf@bcm.edu (M.L.F.); 2 Division of Nutritional Sciences, Cornell University, Ithaca, NY 14853, USA; mac379@cornell.edu; 3 University of Texas Rio Grande Valley School of Medicine, 1210 West Schunior Street, Edinburg, TX 78541, USA; jacob.smith01@utrgv.edu; 4 Department of Pediatrics, Washington University in St. Louis School of Medicine, 660 S. Euclid Ave., St. Louis, MO 63110, USA; manary@kids.wustl.edu

**Keywords:** kwashiorkor, malnutrition, hepatic steatosis, fatty liver disease, choline, betaine, methionine, trimethylamine N-oxide (TMAO), one carbon metabolism, methylation

## Abstract

Hepatic steatosis is a hallmark feature of kwashiorkor malnutrition. However, the pathogenesis of hepatic steatosis in kwashiorkor is uncertain. Our objective was to develop a mouse model of childhood undernutrition in order to test the hypothesis that feeding a maize vegetable diet (MVD), like that consumed by children at risk for kwashiorkor, will cause hepatic steatosis which is prevented by supplementation with choline. A MVD was developed with locally sourced organic ingredients, and fed to weanling mice (*n* = 9) for 6 or 13 days. An additional group of mice (*n* = 4) were fed a choline supplemented MVD. Weight, body composition, and liver changes were compared to control mice (*n* = 10) at the beginning and end of the study. The MVD resulted in reduced weight gain and hepatic steatosis. Choline supplementation prevented hepatic steatosis and was associated with increased hepatic concentrations of the methyl donor betaine. Our findings show that (1) feeding a MVD to weanling mice rapidly induces hepatic steatosis, which is a hallmark disturbance of kwashiorkor; and that (2) hepatic steatosis associated with feeding a MVD is prevented by choline supplementation. These findings support the concept that insufficient choline intake may contribute to the pathogenesis of hepatic steatosis in kwashiorkor.

## 1. Introduction

Undernutrition is a leading cause of child mortality that contributes to the death of more than 1.3 million children every year [1]. The edematous syndrome of kwashiorkor is particularly lethal, with mortality as great as 55% among affected children who require hospitalization [2]. Although undernutrition is typically classified by causal deficiency, i.e., protein-energy or micronutrient deficiencies, the cause of kwashiorkor remains unclear [3]. The defining organ level disturbance of kwashiorkor is severe hepatic steatosis [4], leading early investigators to propose that kwashiorkor be named “infantile fatty liver disease of the tropics” [5]. It was formerly believed that hepatic steatosis in kwashiorkor was due to inadequate protein intake resulting in decreased synthesis of the lipoprotein components of very low density lipoprotein (VLDL), the primary vehicle of lipid export from the liver [6]. However, recent evidence does not support this notion [7], and the cause of hepatic steatosis in kwashiorkor remains uncertain [8]. The inherent complexity of assessing nutritional status in children with kwashiorkor contributes to the uncertainty regarding the nutritional deficiencies that contribute to its pathogenesis. For instance, children with kwashiorkor are often affected by diarrhea, [9], which limits the absorption of essential nutrients that are marginally present in the plant based diets associated with kwashiorkor, such as indispensable amino acids [10,11]. To better investigate the effects of diets associated with kwashiorkor at a mechanistic level, we developed an experimental model produced by feeding weanling mice a maize vegetable diet (MVD), comprised of foods consumed by children with kwashiorkor [12]. This approach is an effort to realistically model the multiple nutritional inadequacies experienced by children at risk for kwashiorkor in a controlled environment, without the additional confounding influence of separate illnesses. In this pilot investigation, we characterized the progressive impact of the MVD on growth and hepatic steatosis, with emphasis on the impact of choline supplementation. The pathogenesis of hepatic steatosis that occurs in association with maize based diets is relevant for understanding the entire kwashiorkor syndrome of malnutrition because hepatic steatosis is its initial hallmark organ level disturbance, which occurs before the onset of the syndromes’ diagnostic feature, edema. Our interest in choline supplementation stems from two observations: that inadequate choline intake is associated with hepatic steatosis [13,14], and that maize-based diets provide little choline [10]. Hepatic steatosis resulting from dietary choline deficiency reflects choline’s essential role in the synthesis of phosphatidylcholine (PC), which is necessary for lipid loading and export of hepatic VLDL [15]. Dietary choline supports PC synthesis by two different pathways: directly through its activation and condensation with diacylglycerol to generate PC in the Kennedy pathway [16], and indirectly through its oxidation to betaine, a methyl donor that supports the synthesis of PC by increasing the availability of methyl groups [17], which are necessary for the methylation of phosphatidylethanolamine to PC catalyzed by phosphatidylethanolamine *N*-methyltransferase (PEMT) [18,19]. Hepatic steatosis in undernutrition may also reflect reduced mTOR signaling, which supports growth and is necessary for phosphatidylcholine and triglyceride secretion [20]. In this pilot investigation, we focused on identifying the metabolic consequences of adding choline to a MVD fed to weanling mice. Specifically, we assessed the influence of choline supplementation on hepatic lipid content, the hepatic concentration of certain metabolites related to the availability and metabolism of methyl (CH_3_) groups, (henceforth referred to collectively as one carbon metabolism), and the hepatic abundance of transcripts related to hepatic lipid export and catabolism: (phosphatidylethanolamine *N*-methyltransferase (PEMT), apolipoprotein-B100 (ApoB100), peroxisome proliferator activated receptor alpha (PPAR-α), and carnitine palmitoyl transferase 1a (CPT1a)).

## 2. Materials and Methods

### 2.1. Murine Diet

A murine diet similar to the weaning diets consumed by children who are at risk for kwashiorkor was constructed using previously published nutritional survey data obtained from caregivers for 239 Malawian children (145 kwashiorkor, 46 marasmus, and 48 healthy controls) [12]. Among the 53 food items surveyed, 7 foods were selected for inclusion in the murine diet (Table 1) based on their predominance in the diets consumed by children with kwashiorkor. USDA certified organic ingredients used to prepare the murine diet were purchased locally in Houston, TX. The boiled maize porridge and vegetable components of the murine diet were prepared separately according to local cooking practices in Malawi. Specifically, organic yellow maize meal (Arrowhead Mills™) was combined with water, brought to a boil, and simmered for 40 min while sugar and non-iodized salt were stirred in [21,22]. Chopped vegetables were boiled for 20 min, drained of excess water, combined with raw mango pulp and bananas, and then homogenized in a commercial blender. The resulting vegetable–fruit purée was blended thoroughly with the cooked maize porridge in a commercial mixer. The resulting homogenized slurry of maize porridge, boiled vegetables, and raw fruit was then stored at −5 °C in one-gallon aliquots prior to lyophilization. Preparation of this novel murine MVD, including lyophilization, was carried out in strict accordance with food industry practices to minimize the risk of bacterial contamination. Samples of the lyophilized diet and control rodent chow were submitted to NP Analytical Laboratories (St. Louis, MO, USA) for nutrient analysis (Table 2 and Table S1). Betaine content was measured separately in laboratories at Cornell University, Ithaca New York.

### 2.2. Animals

Study animals were weanling mice (FVB/N) born to second and third parity dams fed a semi-purified control diet based on AIN 93G [23], (20% protein; Research Diets, New Brunswick, NJ, USA) during gestation and lactation. Weanling mice (11 female and 11 male) were selected for study on post-natal day 21. All experiments were conducted in accordance with the Guide for the Care and Use of Laboratory Animals. The study protocol was approved by the Institutional Animal Care and Use Committee at Baylor College of Medicine.

### 2.3. Animal Protocol

The mice were divided into three separate dietary groups: (1) control chow (CON; Teklad Global Soy Protein-Free Extruded Rodent Diet (2020X), Envigo; *n* = 10); (2) unsupplemented MVD (total *n* = 10); and (3) choline supplemented MVD (MVD + C; *n* = 4). The MVD + C was constructed by adding 1.4 g of choline bitartrate (Hard Eight Nutrition™) to each kg of the MVD, for a total choline concentration of 1.89 g/kg MVD + C diet. This level of choline supplementation was selected due to its similarity to the amount of choline used in prior demonstrations that choline prevents hepatic steatosis in animals subjected to low protein diets [24,25]. In order to assess the progressive effects of feeding the MVD, mice fed the unsupplemented MVD were studied after 6 days (MVD-6; *n* = 5) and 13 days (MVD-13; *n* = 5). MVD + C mice were studied after nine days. CON mice were studied on day 13. Mice were weighed at the beginning and end of the experiment. 

### 2.4. Food Intake

All diets were ground into a powder using the same commercial grade food processor. Food intake for all mice was measured using the same the same monitoring system: all mice were housed individually in monitored units (Comprehensive Laboratory Animal Monitoring System™ (CLAMS) Columbus Instruments, Columbus, OH, USA). This system was used to precisely monitor the amount of food consumed by each mouse while also accounting for spillage, which was collected in a larger tray located beneath the main food cup, which rested on the same balance used to quantify the amount of food removed from the cup. Food and water were available ad libitum throughout the study. Room temperature was maintained at 23.5 °C with a 12 h light/dark cycle. Food intake data was collected using the CLAMS recording system, and together with the energy density values (Table 2), was used to calculate energy intake. Weight adjusted energy intake was calculated by dividing the total Kcal consumed by the animal’s mean body weight and the number of days the animal was studied, i.e., kcal/g BW.day. Daily weight adjusted food intake was calculated similarly using the total weight of food consumed, i.e., g/g BW.day. Feed efficiency was calculated by dividing each animals’ total weight gain by the weight of food consumed throughout the study.

### 2.5. Body Composition

A quantitative magnetic resonance (QMR) analyzer (Echo Medical, Houston, TX, USA) was used to measure total body fat and lean mass on study day one and before euthanasia. This data was used to calculate daily lean and fat mass gain. At the end of the study, mice were anesthetized deeply with isoflurane before decapitation. Hepatic tissue was harvested immediately, snap frozen in liquid nitrogen, and stored at −80 °C until analyzed. The right hepatic lobe of each liver was fixed in formalin, paraffin-embedded, and 4 µm liver sections were cut, processed, and stained with hematoxylin and eosin (H&E) for light microscopy examination following standard procedures. Samples of frozen liver were cryosectioned (10 µM) onto glass slides, fixed in 10% formaldehyde, and stained for lipid with Oil Red O, followed by H&E and a final immersion in toluidine blue and basic fucsin stain before mounting in aqueous glycerin jelly media. Photometric assessment of the amount of Oil Red O stained lipid material present in liver tissue was performed using a customized MATLAB™ algorithm. Briefly, digital images of liver tissue were converted into a MATLAB™ format consisting of three separate color spectrums: red, grey, and blue. Each pixel was then cross referenced with a reference histologic material; (i.e., lipid, (red), vacant background (grey), or nucleic material, (blue)), which allowed quantification of lipid droplets in each liver tissue specimen. Periodic acid Schiff (PAS) staining of liver tissue for light microscopy was undertaken using 5 µm sections of liver tissue that were first deparaffinized and hydrated in water before placement in 0.5% periodic acid solution for 5 min. Tissue sections were then rinsed in distilled water and placed in Schiff reagent for 15 min before rinsing in tap water. Sections were counterstained in Mayer’s hematoxylin for one minute, dehydrated in alcohol, and then covered using a synthetic mounting medium. Histologic examination of liver tissue specimens were conducted in the pathology laboratories at Texas Children’s Hospital, Houston, Texas, by a pathologist who remained blinded to the dietary treatments.

### 2.6. Metabolite Concentrations

Liver samples from MVD-fed mice (MVD-6, *n* = 4; MVD-13, *n* = 4), MVD + C mice (*n* = 3), and CON mice (*n* = 3) were available for further metabolite and transcriptional analyses. To measure the concentration of methionine and choline-related metabolites in liver, samples (0.02–0.05 g) were homogenized (Argos Technologies Pestle Motor Mixer A0001) in 400 µL of 2:1 methanol/chloroform. Metabolite concentrations in the liver homogenates and in-house controls were measured using established LC–tandem MS methods reported previously [23], with minor protocol adaptations allowing use of local instrumentation. All metabolite measurements were made on the same day using the same LC-tandem MS machinery and setup. Homogenized liver tissue specimens from healthy adult mice with defined metabolite concentrations were used as quality control samples. The assay imprecision (i.e., coefficient of variation), was <5% for betaine, free choline, and dimethylglycine, and 8.8% for methionine. All metabolite data were generated in laboratories at Cornell University, Ithaca, New York.

### 2.7. mRNA Quantification by Quantitative Reverse-Transcriptase (RT) PCR

RNA was extracted from frozen liver samples from MVD (MVD-6, *n* = 3; MVD-13, *n* = 3), MVD + C (*n* = 3), and CON mice (*n* = 3). Total RNA was extracted with Trizol reagent (Invitrogen™, Carlsbad, CA, USA) using the manufacturer’s protocol. RNA concentration and quality were assessed using a NanoDrop ND-1000 spectrophotometer. cDNA was generated using a high capacity cDNA reverse transcription kit (Applied Biosystems, Foster City, CA, USA, Thermo Fisher Scientific, Waltham, MA, USA) and an Eppendorf 5331 Mastercycler. Quantitative PCR was performed with a BioRad C-1000 Touch Thermal Cycler using SYBR Green Supermix reagents (Bio-Rad Laboratories, Hercules, CA, USA). Primers used in this investigation (i.e., ß-glucuronidase [Gusb], PEMT, ApoB, PPAR-α, and CPT1a), were identified from the literature and confirmed using the NCBI Primer-BLAST algorithm. Forward and reverse primer sequences are shown in Supplementary Table S2. Primer efficiencies were calculated (1.8–2.2) following amplification of a standard curve. Melting curves were included at the end of amplification cycles to validate specificity of the PCR product. The 2^−ΔCt^ method was used to calculate fold changes normalized to the expression of the housekeeping gene, β-glucuronidase (Gusb).

### 2.8. Statistical Analyses

The normality and homogeneity of generated data were confirmed using a Kolmogorov–Smirnov test [26] and Levene’s test [27], respectively. Due to group size asymmetry, a Welch’s ANOVA [28] was paired with a Tukey honest significant difference (HSD) [29] post hoc analysis with a confidence level of 0.95 (*p* < 0.05) in order to measure intergroup differences for feeding and metabolite data. Statistical analysis of transcriptional data was performed using R (version 3.4.0), with differences assessed using a standard one-way ANOVA. Data are presented as means ± SE.

## 3. Results

### 3.1. Comparison of Study Diets

Although the energy density of the MVD was nearly identical to that of regular chow, macronutrient analysis demonstrated that the MVD contained a disproportionately larger amount of carbohydrate at the expense of protein and fat. Specifically, the MVD contained 35, 49, and 135% of the protein, fat, and carbohydrate present in regular chow, respectively (Table 2). The concentration of all measured minerals and micronutrients, with the exception of sodium, were significantly lower in the MVD (Table S1). The sodium content of the MVD is a reflection of the addition of non-iodized salt during preparation of the MVD, in accordance with prevailing culinary practices in Sub-Saharan Africa [24]. Consistent with the reduced protein content of the MVD, the concentration of individual amino acids in the MVD ranged from 22% to 43% of the amount present in the regular chow diet. With regard to the comparative content of nutrients that support one carbon metabolism, the MVD provided 22% of the methionine content, 30% of the choline content, 26% of the betaine content, and 6% of the folate content of regular chow.

### 3.2. Weight Gain and Feed Intake

**Figure 1:**
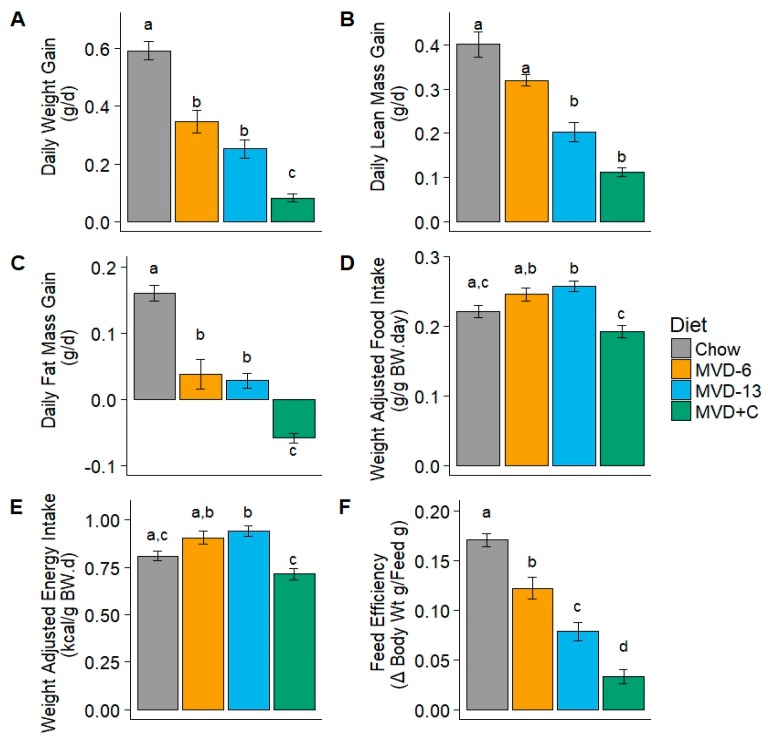
Weight gain, composition of weight gain, and food intake parameters of weanling mice fed a control chow diet (*n* = 10), the maize vegetable diet (MVD) for 6 days (MVD-6; *n* = 5) or 13 days (MVD-13; *n* = 5), or 9 days with a MVD supplemented with choline (MVD + C; *n* = 4). (**A**) daily weight gain; (**B**) daily lean mass gain; (**C**) daily fat mass gain; (**D**) daily weight-adjusted food intake; (**E**) daily weight-adjusted energy intake; and (**F**) feed efficiency. Presence of a statistically significant difference between data points (*p* < 0.05) is designated by the absence of a shared lowercase letter over the data point. Bars represent mean values ± SE.

Compared to CON mice, MVD fed mice had lower daily weight gain, (*p* < 0.01; Figure 1A), with MVD + C mice exhibiting the most pronounced effect. Daily lean gain was significantly lower in all MVD-fed mice compared to CON; (*p* < 0.01; Figure 1B). All three MVD diets exhibited lower daily fat mass gain (*p* < 0.05; Figure 1C), than mice fed CON chow. This effect was most pronounced in MVD + C mice, which lost total fat mass despite gaining total weight and lean mass. In MVD6 and MVD13 groups of mice there was a largely non-significant trend of weight adjusted food and energy intake that was greater than that of control mice, whereas in MVD + C mice there was a non-significant opposite trend (Figure 1D,E). All MVD-fed mice had feed efficiencies that were significantly less than that of controls (Figure 1F). This effect was most pronounced in MVD + C mice.

### 3.3. Histology

**Figure 2:**
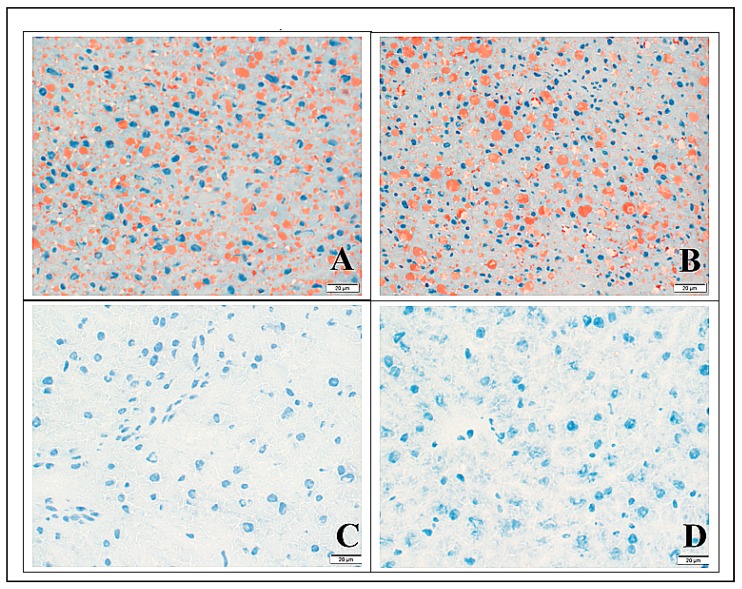
Oil Red O stained liver tissue (60×, scale bar represents 20 µm) collected from mice fed maize vegetable diet (MVD) diets or chow. Red staining areas indicate the presence of fat; dark blue staining indicates nuclear material*.* (**A**) Liver section from mouse fed the unsupplemented MVD for 6 days; (**B**) liver section from mouse fed the unsupplemented MVD for 13 days; (**C**) liver section from mouse fed the MVD supplemented with choline for 9 days; and (**D**) liver section from a control mouse fed chow for 13 days.

**Figure 3:**
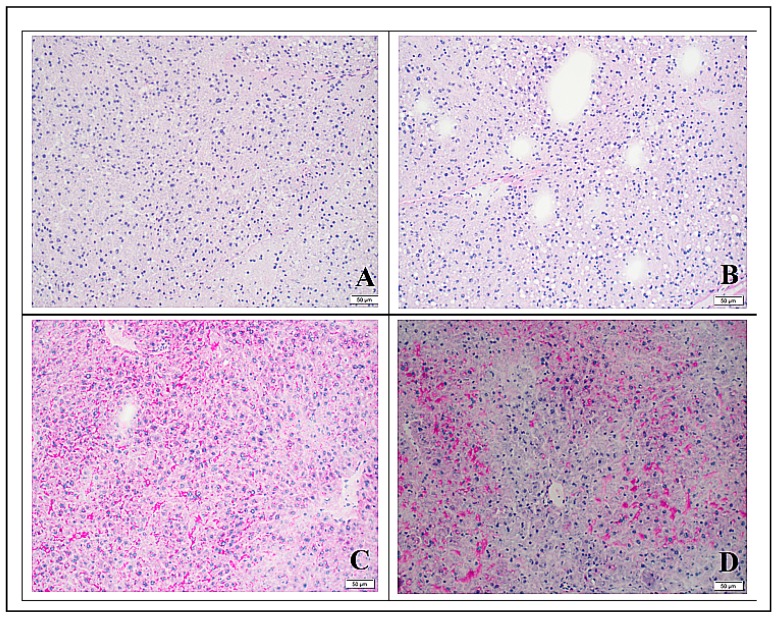
Periodic acid Schiff (PAS) stained liver tissue (20×, scale bar represents 20 µm) collected from mice fed maize vegetable diet (MVD) diets or chow. Bright red staining areas indicate the presence of polysaccharides; dark blue staining indicates nuclear material. (**A**) Liver section from mouse fed the unsupplemented MVD for 6 days; (**B**) liver section from mouse fed the unsupplemented MVD for 13 days; (**C**) liver section from mouse fed the MVD supplemented with choline for 9 days; and (**D**) liver section from a control mouse fed chow for 13 days.

The most prominent finding was noted on microscopic examination of Oil Red O stained liver tissue, which demonstrated the pronounced accumulation of lipids in hepatocytes in MVD-6 and MVD-13 mice (relative to chow fed mice) (Figure 2A,B,D). The absence of this phenotype in MVD + C mice (Figure 2C) is consistent with our hypothesis that choline supplementation prevents the development of hepatic steatosis in weanling mice subjected to a MVD. Quantitative assessment of hepatic lipid depositions using photometric tools confirmed the statistical significance of this observation (Table S3). Direct comparison of lipid localization within the three separate zones of the portal triad in MVD-fed mice did not reveal a specific distribution pattern. Rather, lipid droplets were located evenly throughout the three zones of the hepatic lobule. Although there were significant accumulations of hepatic lipids in mice fed the unsupplemented MVD, there was no evidence of inflammation on H&E sections. PAS stained liver tissue from MVD + C mice (Figure 3C) was not different CON mice (Figure 3D), with a similar abundance and distribution of PAS stained material located primarily in zone three. Liver tissue from both groups of mice fed an unsupplemented MVD (Figure 3A,B) was nearly free of PAS stained material.

### 3.4. Liver Metabolites

**Figure 4:**
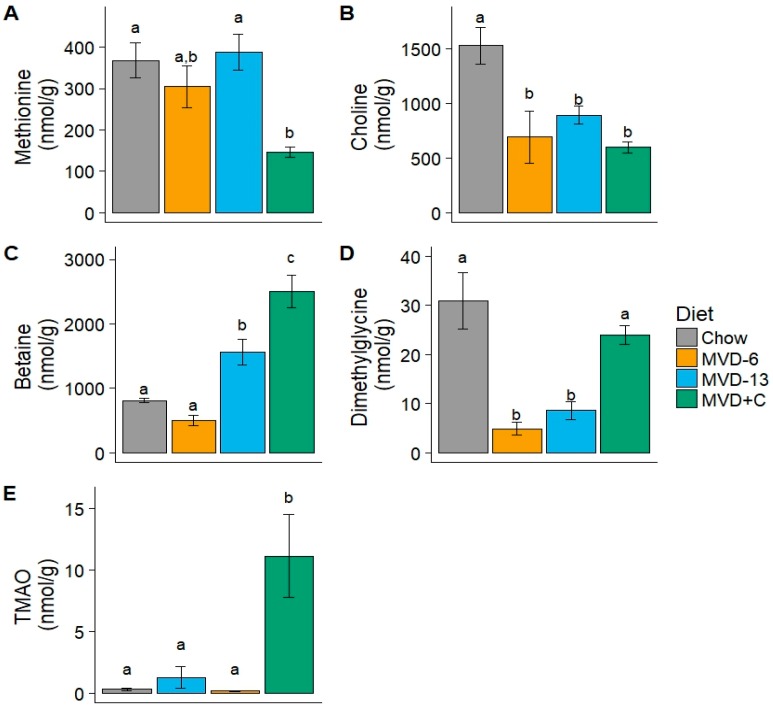
Hepatic concentration of metabolites in mice fed the control chow diet (*n* = 3), maize vegetable diet (MVD) for 6 days (MVD-6; *n* = 4) or 13 days (MVD-13; *n* = 4), or MVD with supplemental choline for 9 days (MVD + C; *n* = 3): (**A**) methionine; (**B**) choline; (**C**) betaine; (**D**) dimethylglycine; (**E**) trimethylamine N-oxide (TMAO). The presence of statistically significant differences among the dietary regimens (*p* < 0.05), is designated by the absence of a shared lowercase letter over the data point. Bars represent mean values ± SE.

Quantification of the hepatic concentration of methionine, choline, and the choline-related metabolites betaine and dimethylglycine, provided insight into the hepatic metabolism of choline in the different groups of mice. Whereas the mean hepatic concentration of methionine was similar in CON, MVD-6, and MVD-13 groups, hepatic methionine was significantly lower in MVD + C mice (Figure 4A and Table S3). Hepatic choline was significantly lower in all MVD fed mice, including MVD + C, relative to chow (Figure 4B), whereas hepatic betaine (produced from the oxidation of choline [30]), was highest in mice fed the MVD + C diet (*p* < 0.05; Figure 4C). Hepatic dimethylglycine, which is produced from the demethylation of betaine, was lower (*p* < 0.05) in both groups of mice fed an unsupplemented MVD, to both chow and MVD + C mice (Figure 4D). Trimethylamine N-oxide (TMAO), a plasma metabolite which is increased when dietary choline is metabolized by the gut microbiome [25], was significantly elevated in MVD + C mice (*p* < 0.05; Figure 4E).

### 3.5. Transcriptional Targets

The results of RT-qPCR analyses were notable for a trend of greater transcription of ApoB (*p* = 0.059), in MVD + C mice (Figure S1).

## 4. Discussion

Despite significant research into the pathogenesis of kwashiorkor, there is not a consensus regarding the cause of this syndrome’s characteristic hepatic steatosis [3]. Although formerly believed to be due to inadequate protein intake, it is now known that this alone is not sufficient to precipitate this hallmark disturbance of kwashiorkor [31]. An undernourished mouse model that recapitulates the defining organ level disturbance of kwashiorkor, hepatic steatosis, holds potential to offer new insights for understanding the nutritional deficiencies that contribute to the pathogenesis of this poorly understood syndrome of childhood undernutrition. To address this need we used previously obtained nutritional survey data [12] to develop a murine diet comprised of food items commonly consumed by children who are at increased risk for kwashiorkor. This novel murine diet was readily consumed, as evidenced by the greater weight-adjusted intake of the unsupplemented diet in MVD-13 mice, relative to CON mice. Weight adjusted intake of the choline supplemented diet was lower than that of MVD-13 mice, but not significantly different from CON and MVD-6 groups. We speculate that lower weight adjusted food intake in MVD + C mice was due to the sour taste of the choline bitartrate used to supplement the MVD, which may have inadvertently reduced the palatability of the MVD. Not unexpectedly, MVD-fed mice had significantly lower daily weight gain than mice fed the control chow (Figure 1). This is consistent with the observation that plant-based diets low in protein and micronutrients result in growth stunting despite adequate energy content [32]. The pattern of reduced total weight gain in MVD-fed mice relative to CON mice was reflected by both lower lean and fat mass gains. The lower weight gain of MVD + C mice relative to mice in the unsupplemented MVD groups was attributable to decreased fat gain. This observation corresponds with recent demonstrations that choline deficiency is associated with impaired lipid metabolism [26], and that choline supplementation promotes molecular shifts associated with increased fat catabolism [27,28,29,33]. However, decreased fat gain was not observed in mice consuming the control diet, which also provided ample amounts of choline, suggesting that the metabolic effects of choline is unique in the context of the MVD. The observation of reduced fat gain in MVD + C mice warrants further exploration in studies utilizing stable isotopes and systems biology approaches.

The primary hypothesis of this investigation was that feeding weanling mice a diet similar to that consumed by children at risk for kwashiorkor will result in hepatic steatosis, which is a hallmark pathophysiologic feature of kwashiorkor [30]. We documented that mice fed the unsupplemented MVD developed significant hepatic steatosis within six days of consuming the MVD (Figure 2). There was no evidence of steatohepatitis, an inflammatory sequelae of nonalcoholic fatty liver disease (NAFLD) that is closely correlated with the duration of steatosis. This is likely a reflection of the relatively short time-frame that mice were exposed to the steatogenic unsupplemented MVD. This pattern of hepatic steatosis corresponds with the findings of a similarly constructed porcine model of malnutrition [34]. We also demonstrated that PAS stained hepatic tissue from mice that were fed an unsupplemented MVD had reduced quantities of PAS staining material relative to CON mice, consistent with a limited presence of glycogen. This pattern of PAS staining reflects of the complex metabolic disturbances precipitated by the MVD, which also reduced hepatic glycogen stores.

**Figure 5:**
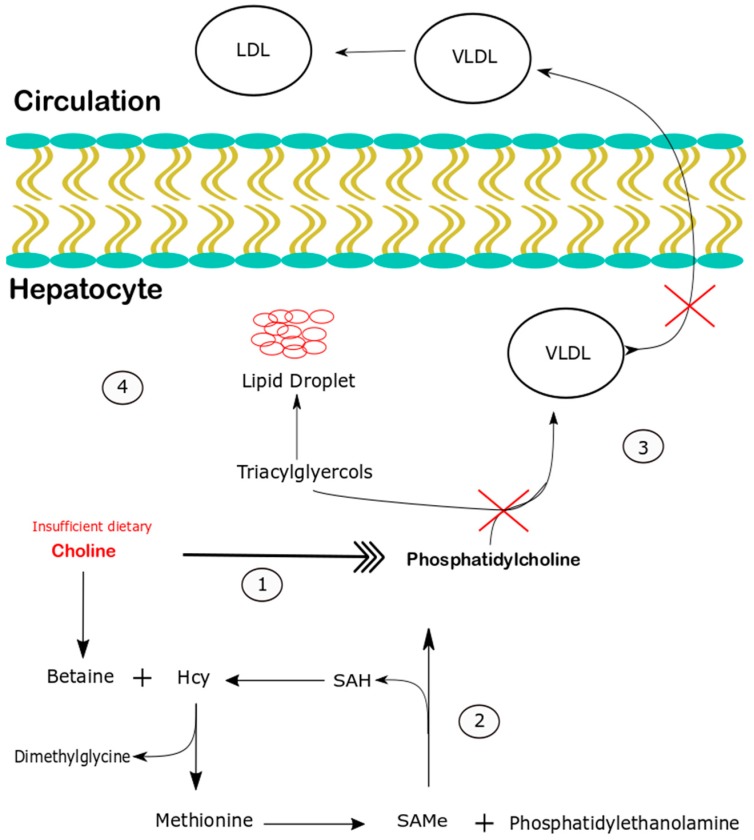
Dietary choline supports the production of phosphatidylcholine (PC) and VLDL export from hepatocytes through two pathways; (**1**) Choline provides direct support for the CDP-choline pathway, in which choline is incorporated into the choline headgroup of PC, and (**2**) Choline indirectly supports PC synthesis by the phosphatidylethanolamine *N*-transferase pathway, in which choline is oxidized to betaine, a methyl donor that increases the availability of labile methyl groups for the trimethylation of phosphatidylethanolamine to PC. (**3**) PC produced through the PEMT and CDP-choline pathways supports the formation and secretion of VLDL. (**4**) When choline intake is insufficient to maintain PC synthesis, VLDL synthesis and export are impaired (indicated by red hash marks), resulting in triacylglycerol accumulations in the form of lipid droplets.

Additional efforts were directed towards understanding the role of one carbon metabolism disturbances in the pathogenesis of hepatic steatosis precipitated by feeding the MVD. One carbon metabolism disturbances are often associated with NAFLD due to the fact that loading of triglycerides into VLDL and the hepatic secretion of VLDL require phosphatidylcholine (PC) synthesized by the methylating action of PEMT [35]. The association between NAFLD and one carbon metabolism disturbances is relevant for understanding the pathogenesis of hepatic steatosis in undernutrition because inadequate intake of nutrients essential for one carbon metabolism often leads to decreased hepatic PC synthesis by the PEMT pathway, resulting in hepatic steatosis as a consequence [36,37] (Figure 5). Notably, the MVD contained smaller quantities of several nutrients that are essential for maintaining the availability of labile one-carbon molecules (Table 2). In addition to containing only a third as much methionine as control chow, the MVD provided smaller quantities of the methyl donors, folate and betaine, as well as the essential precursor of betaine, choline. This pattern of nutrient depletion in the MVD is similar to that of experimental diets designed to precipitate hepatic steatosis [38,39].

Although it is known that choline supplementation prevents hepatic steatosis associated with established low protein diet models of undernutrition [40], it was not known whether increased choline intake would alone be sufficient to prevent hepatic steatosis associated with an MVD, which is deficient in multiple nutrients essential for one carbon metabolism. Therefore, we chose to focus on the metabolic fate of supplemental choline in this initial investigation of the MVD. Although it is clear that the nutritional deficiencies that cause kwashiorkor are more complex than simple choline deficiency, a greater understanding of choline’s role in the pathogenesis of hepatic steatosis associated with kwashiorkor carries the potential to guide the development of strategies to prevent kwashiorkor in children. The finding that MVD + C mice had significantly greater hepatic concentrations of betaine, but not choline (Figure 4C), suggests that supplemental choline was readily oxidized to the methyl donor betaine. Similarly, hepatic concentrations of dimethylglycine, which is formed from the demethylation of betaine, were higher in MVD + C mice, compared to both unsupplemented MVD groups (Figure 4D), indicating that choline supplementation increased the hepatic availability of methyl groups. Notably, increased hepatic availability of the methyl donor betaine in MVD + C mice was associated with significantly lower hepatic concentrations of methionine (Figure 4A). Although methionine is critical for the formation of the universal methyl donor S-adenosylmethionine [41], which is necessary for PEMT activity [42] and VLDL export [18] from hepatocytes (Figure 5), reduced hepatic methionine in MVD + C mice was not associated with steatosis. The demonstration of decreased hepatic methionine without steatosis suggests that choline supplementation promoted more efficient methionine use in the context of the MVD.

Our demonstration of greater hepatic TMAO concentrations in choline supplemented mice (Figure 4E) is consistent with the fact that increased choline intake leads to increased availability of choline for metabolism by the gut microbiome, thus promoting the formation of TMAO [43]. Although increased TMAO has been associated with hepatic steatosis in humans [44], this was not the case in this investigation. Rather, MVD + C mice, which had the greatest concentrations of hepatic TMAO, did not develop steatosis. This finding is notable given the well described association between increased plasma TMAO and the risk for various metabolic disease states [45] closely associated with hepatic steatosis and suggests that increased TMAO does not by itself cause the pathogenesis of hepatic steatosis in undernutrition. The observation that TMAO levels were increased in the MVD + C mice but not chow mice, which consumed similar levels of choline, suggests that the short term feeding of the MVD altered TMAO handling. Future studies examining the composition of the gut microbiome, kinetics of TMAO metabolism, influence of TMAO on hepatic inflammation, as well as urinary and fecal excretion of TMAO, are warranted.

The observed trend of greater apolipoprotein-B100 transcription in mice consuming the MVD + C diet (*p* = 0.059), suggests that choline supplementation also supported greater synthesis of apolipoprotein-B100, an essential component of VLDL, which is the primary vehicle of lipid export from the liver [46]. However, larger sample sizes are necessary to precisely characterize the molecular effects of choline supplementation in this novel undernourished mouse model.

Our study was constrained by limitations that are important to consider. While the obtained metabolic data are relevant to hepatic PC synthesis, the small quantity of hepatic tissue available for analyses in this initial investigation limited our ability to quantify PC and PC subspecies. Similarly, the interpretability of transcriptional data was limited by the small number of tissue specimens available for analysis in this initial study, and also by the fact that transcriptional content is not necessarily a reliable proxy for the amount of protein translated. In addition to supporting PC synthesis through the PEMT pathway, choline also supports the direct synthesis of PC by the cytidine diphosphate (CDP)-choline pathway [16]. Although increased CDP-choline pathway activity is likely to have been metabolically relevant in MVD + C mice, accurate measurement of CDP activity requires stable isotope techniques that are beyond the scope of this initial study. Larger studies using isotopically labeled choline [47] are likely to provide more precise insight for understanding the predominant mechanism by which choline supplementation prevented hepatic steatosis in this novel mouse model of undernutrition.

## 5. Conclusions

In summary, feeding weaning mice a diet composed primarily of maize and vegetables that is similar to the diets consumed by children at risk for kwashiorkor resulted in hepatic steatosis, a defining organ level disturbance of kwashiorkor [30]. Notably, hepatic steatosis developed in as little as six days and was prevented by choline supplementation alone, without supplementing with other essential one carbon nutrients such as folate or methionine, which like choline were minimally present in the MVD. Metabolic data suggested that the prevention of hepatic steatosis in mice fed a choline-supplemented MVD was associated with a significant increase in the hepatic availability of methyl groups. This finding is consistent with the observation that VLDL export from the liver has an absolute requirement for PC synthesized by PEMT [35,48] a methyl group dependent enzyme. Overall, these findings provide insight into the molecular mechanisms influenced by the addition of choline to a MVD, and highlight the potential role of one-carbon nutrients, such as choline, for preventing hepatic steatosis in children subsisting on low protein plant based diets that are associated with increased risk for kwashiorkor malnutrition.

## Figures and Tables

**Table 1:** Ingredient composition of the maize-vegetable diet (MVD) before lyophilization.

Food Ingredient	Content (g/kg)
Maize meal	300
Peeled onions	105
Mustard greens	135
Turnip greens	135
Mangos	90
Bananas	65
Non-iodized salt	15
Raw cane sugar	155

**Table 2:** Nutrient composition * of lyophilized maize vegetable diet and Teklad 2020X™ control chow.

	Maize Vegetable Diet	Control Chow	MVD% of Control Chow
Metabolizable Energy, kcal/kg	3693	3683	100.3
Protein, g/kg	64	183	35.0
Carbohydrate, g/kg	784	581	134.9
Fat, g/kg	34	71	47.9
Fiber, g/kg	15	28	53.6
Ash, g/kg	40	41	97.6
Moisture, g/kg	64	97	66.0
One Carbon Micronutrients and Sulfur Amino Acids			
Choline, mg/kg	489	1620	30.2
Betaine, mg/kg	240	900	26.7
Folic Acid, mg/kg	0.15	2.46	6.1
Pyridoxine, mg/kg	2.46	11.9	20.7
Cysteine, mg/kg	1150	3700	31.1
Methionine, mg/kg	1020	4600	22.2

* Analyzed by NP Analytical Laboratories, St. Louis, Missouri.

## Supplementary Materials

The following are available online at http://www.mdpi.com/2072-6643/10/5/653/s1: Table S1: Mineral and amino acid content of the Maize Vegetable Diet after lyophilization and the Teklad 2020X™ Control Chow; Table S2: Forward and reverse sequences of primers used to measure the concentration of mRNA in liver tissue; Table S3: Baseline characteristics, growth data, and hepatic concentrations of measured metabolites for different groups of weanling mice fed different diets; Maize Vegetable Diet fed for 6 days, (MVD-6), Maize Vegetable Diet fed for 13 days (MVD-13), Maize Vegetable Diet with supplemental choline, for 9 days (MVD+C), and control Chow for 13 days; Figure S1: Hepatic concentrations of mRNA transcripts in weanling mice fed the control chow diet (*n* = 3), the maize vegetable diet (MVD) for 6 days (MVD-6; *n* = 4) or 13 days (MVD-13; *n* = 4), or the MVD with supplemental choline for 9 days (MVD + C; *n* = 3).
