# Supplementary material for: Choline Supplementation Prevents a Hallmark Disturbance of Kwashiorkor in Weanling Mice Fed a Maize Vegetable Diet: Hepatic Steatosis of Undernutrition
Source: Nutrients. 2018 May 22;10(5):653. doi: 10.3390/nu10050653 (PMC5986532; doi:10.3390/nu10050653)
Supplement: Supplementary file 1 [file nutrients-10-00653-s001.pdf]

**Supplemental Table 1:** Mineral and amino acid content of the Maize Vegetable Diet after lyophilization and the Teklad 2020X™ Control Chow*		
	Maize Vegetable Diet	Teklad 2020™ Control Chow
Minerals		
Sodium mg/kg	11510	1396
Potassium mg/kg	3302	4196
Calcium mg/kg	835	8579
Phosphorous mg/kg	1963	6782
Magnesium mg/kg	783	2433
Iron mg/kg	45	191
Zinc mg/kg	18	56.4
Copper mg/kg	3	12.0
Chromium mg/kg	<1	6.68
Arsenic mg/kg	<0.05	0.11
Selenium mg/kg	0.14	0.36
Cadmium mg/kg	<0.05	<0.05
Lead mg/kg	0.22	0.1
Manganese mg/kg	11	77
Amino Acids		
Aspartic Acid mg/kg	4400	11100
Threonine mg/kg	2200	5900
Serine mg/kg	2800	8500
Glutamic Acid mg/kg	11500	41900
Proline mg/kg	5100	15900
Glycine mg/kg	2400	6300
Alanine mg/kg	4400	12500
Valine mg/kg	3100	8500
Isoleucine mg/kg	2200	6600
Leucine mg/kg	6900	21500
Tyrosine mg/kg	1700	6400
Phenylalanine mg/kg	3100	9800
Histidine mg/kg	1700	3900
Lysine mg/kg	1900	7800
Arginine mg/kg	2800	7400
Tryptophan mg/kg	<500	1710
Cysteine mg/kg	1150	3700
Methionine mg/kg	1020	4600
**Analyses of both diets were conducted by NP Analytic Laboratories, St. Louis Missouri*		

**Supplemental Table 2:** Forward and reverse sequences of primers used to measure the concentration of mRNA in liver tissue				
Gene	*Sequence (5′->3′)*	*Length*	*Tm*	*Slope*
**Phosphatidylethanolamine N-Methyltransferase**				-3.188
Forward primer	ACTCATGCATGCTAGTCCCA	20	58.79	
Reverse primer	AGCAGTGAAGGGCTCTTCAT	20	59.01	
Apolipoprotein B				-3.798
Forward primer	TTGGCAAACTGCATAGCATCC	21	59.52	
Reverse primer	TCAAATTGGGACTCTCCTTTAGC	23	58.41	
**Peroxisome Proliferator Activated Receptor Alpha**				-3.49
Forward primer	AGAGCCCCATCTGTCCTCTC	20	60.4	
Reverse primer	ACTGGTAGTCTGCAAAACCAAA	22	58.37	
**Carnitine Palmitoyltransferase 1 Alpha**				-3.423
Forward primer	CTCCGCCTGAGCCATGAAG	19	60.52	
Reverse primer	CACCAGTGATGATGCCATTCT	21	58.35	
**Beta Glucuronidase**				-3.322
Forward primer	CCGACCTCTCGAACAACCG	19	60.44	
Reverse primer	GCTTCCCGTTCATACCACACC	21		

Supplemental Table 3: Baseline characteristics, growth data, and hepatic concentrations of measured metabolites for different groups of weanling mice fed different diets; Maize Vegetable Diet fed for 6 days, (MVD-6), Maize Vegetable Diet fed for 13 days (MVD-13), Maize Vegetable Diet with supplemental choline, for 9 days (MVD+C), and control Chow for 13 days. Values are means ± SE; n = number of liver tissue specimens available for analysis.

	**MVD6**	**P Value**			**MVD13**	**P Value**		**MVD+C**	**P Value**	**Chow**
		**MVD6 vs. MVD13**	**MVD6 vs. MVD+C**	**MVD6 vs. Chow**		**MVD13 vs. MVD+C**	**MVD13 vs. Chow**		**MVD+C vs. Chow**	
Total number of mice / Number of female mice	4 / 1				5 / 2			4 / 2		10 / 6
Start Weight Body Wt. (g) on PND 21	10.47 ± *0.08*	0.91	0.014	0.06	10.86 ± *0.2*	0.04	0.17	12.59 ± *0.18*	0.55	11.9 ± *0.39*
Mean Daily Wt. Gain g/day	0.35 ± 0.08	0.36	<0.01	<0.01	0.25 ± 0.07	0.03	<0.01	0.08 ± 0.03	<0.01	0.59 ± 0.03
Mean Daily Lean Wt. Gain g/day	0.32 ± 0.03	0.08	<0.01	0.21	0.2 ± 0.02	0.22	<0.01	0.11 ± 0.01	<0.01	0.4 ± 0.03
Mean Daily Fat Wt. Gain g/day	0.04 ± 0.02	0.97	<0.01	<0.01	0.03 ± 0.01	<0.01	<0.01	-0.06 ± 0.01	<0.01	0.16 ± 0.01
Wt. Adjusted Food intake g / Bw.day	0.25 ± 0.01	0.87	0.01	0.26	0.26 ± 0.01	<0.01	0.04	0.19 ± 0.01	0.16	0.22 ± 0.01
Wt. Adjusted Energy Intake Kcal / g BW.day	0.91 ± 0.03	0.89	0.01	0.15	0.94 ± 0.03	<0.01	0.02	0.72 ± 0.03	0.16	0.81 ± 0.03
Feed Efficiency Δ Wt. g / g Feed	0.12 ± 0.01	0.02	<0.01	<0.01	0.08 ± 0.01	0.02	<0.01	0.03 ± 0.01	<0.01	0.17 ± 0.01
Hepatic Methionine nmol/g	305 ± 50 n=3	0.49	0.11	0.729	389 ± 43 n=4	0.01	0.982	147 ± 12 n=3	0.02	368 ± 43 n=3
Hepatic Choline nmol/g	693 ± 238 n=4	0.74	0.97	0.01	895 ± 79 n=4	0.48	0.04	598 ± 51 n=3	0.01	1532 ± 171 n=3
Hepatic Dimethylglycine nmol/g	4.89 ± 1.28 n=4	0.81	0.01	<0.01	8.61 ± 1.85 n=4	0.02	0.02	24.0± 2.0 n=3	0.44	31.00 ± 5.73 n=3
Hepatic Betaine nmol/g	501 ± 78 n=4	0.01	<0.01	0.64	1566 ± 198 n=4	0.02	0.05	2502 ± 252 n=3	<0.01	812 ± 31 n=3
Hepatic TMAO nmol/g	1.25 ± 0.86 n=4	0.96	0.01	0.97	0.15 ± 0.03 n=4	<0.01	1.0	11.17 ± 3.4 n=3	<0.01	0.29 ± 0.09 n=3
Relative PEMT Expression	3.34 ± 1.3 n=3	0.5	0.73	0.26	0.28 ± 0.08 n=3	0.14	0.96	5.53 ± 2.63 n=3	0.26	1.28 ± 0.44 n=3
Relative CPT1a Expression	3.55 ± 1.95 n=3	0.93	1.0	0.99	1.03 ± 0.37 n=3	0.06	0.79	14.11 ± 5.52 n=3	0.2	4.91 ± 0.4 n=3
Relative Apo B100 Expression	2.68 ± 1.05 n=3	0.98	1.0	0.26	0.58 ± 0.21 n=3	0.41	0.39	2.41 ± 1.09 n=3	0.48	1.61 ± 0.41 n=3
Relative PPAR α Expression	0.43 ± 0.08 n=3	0.98	0.86	0.18	0.83 ± 0.35 n=2	0.99	0.99	1.18 ± 0.29 n=3	0.48	2.63 ± 1.21 n=3
Hepatic Fat % Objects staining red in liver	0.72 ± 0.01 n=3	0.99	0.01	0.01	0.71 ± 0.01 n=2	0.03	0.03	0.18 ± 0.12 n=4	1.0	0.183 ±0.1 n=4

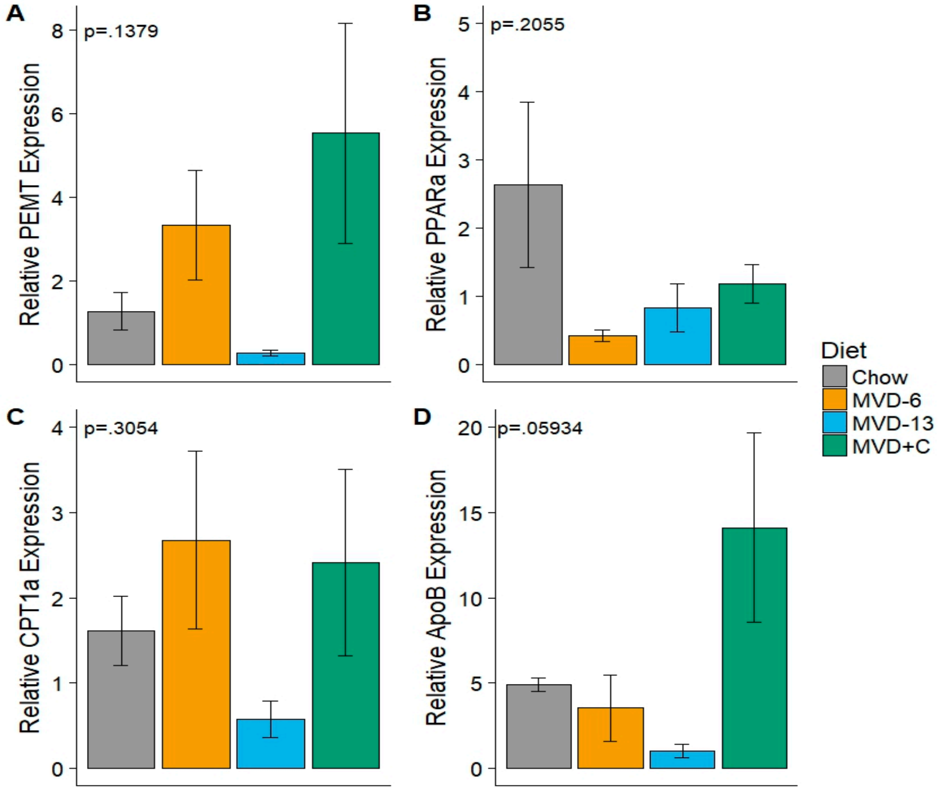

Figure S1. Hepatic concentrations of mRNA transcripts in weanling mice fed the control chow diet ($n = 3$), the maize vegetable diet (MVD) for 6 days (MVD-6; $n = 4$) or 13 days (MVD-13; $n = 4$), or the MVD with supplemental choline for 9 days (MVD + C; $n = 3$). (**A**) phosphatidylethanolamine methyltransferase (PEMT); (**B**) peroxisomal proliferator activated receptor-α (PPAR-α); (**C**) carnitine palmitoyl transferase 1a (CPT1a); and (**D**) apolipoprotein-B100 (ApoB). Values are expressed as $2^{\Delta Ct}$ relative to ß-glucuronidase. Bars represent mean values ± SE.
